# Lassa Fever among Children in Eastern Province, Sierra Leone: A 7-year Retrospective Analysis (2012–2018)

**DOI:** 10.4269/ajtmh.20-0773

**Published:** 2020-11-23

**Authors:** Robert J. Samuels, Troy D. Moon, Joseph R. Starnes, Foday Alhasan, Michael Gbakie, Augustine Goba, Veronica Koroma, Mambu Momoh, John Demby Sandi, Robert F. Garry, Emily J. Engel, Jeffrey G. Shaffer, John S. Schieffelin, Donald S. Grant

**Affiliations:** 1 Vanderbilt Institute for Global Health, Vanderbilt University Medical Center, Nashville, Tennessee;; 2 Lassa Fever Program, Kenema Government Hospital, Ministry of Health and Sanitation, Kenema, Sierra Leone;; 3 Department of Immunology and Microbiology, School of Medicine, Tulane University, New Orleans, Louisiana;; 4 Department of Pediatrics and Internal Medicine, Sections of Pediatric and Adult Infectious Diseases, School of Medicine, Tulane University, New Orleans, Louisiana;; 5 Department of Global Biostatistics and Data Science, School of Public Health and Tropical Medicine, Tulane University, New Orleans, Louisiana;; 6 College of Medicine and Allied Health Sciences, University of Sierra Leone, Freetown, Sierra Leone

## Abstract

Pediatric Lassa fever (LF) usually presents as a nonspecific febrile illness, similar to other endemic diseases in countries like Sierra Leone, where LF is considered to be hyperendemic. The nonspecificity of presentation and lack of research have made it difficult to fully understand best practices for pediatric management. We aim to describe clinical characteristics of hospitalized pediatric patients suspected or diagnosed with LF and assess factors associated with hospital outcomes among those with LF antigen–positive results. We conducted a 7-year retrospective cohort study using routine data for all children younger than 18 years admitted at the Kenema Government Hospital’s LF ward. A total of 292 children with suspected or confirmed LF were analyzed. Overall, mortality was high (21%). Children with antigen-positive results had a high case fatality rate of 63% (*P* < 0.01). In univariate analyses, children who presented with unexplained bleeding (odds ratio [OR]: 3.58; 95% CI: 1.08–11.86; *P* = 0.040) and confusion (altered sensorium) (OR: 5.37; 95% CI: 1.34–21.48; *P* = 0.020) had increased odds of death. Abnormal serum levels of alanine aminotransferase (*P* = 0.001), creatinine (*P* = 0.004), and potassium (*P* = 0.003) were associated with increased likelihood of death in these children. Treatment with ribavirin was not significantly associated with survival (*P* = 0.916). Our findings provide insights into current pediatric LF clinical presentation and management. More evidence-based, high-quality research in creating predictive algorithms of antigen-positivity and hospital outcomes is needed in the management of pediatric LF.

## INTRODUCTION

Lassa fever (LF) is endemic in at least nine countries in West Africa.^1^ Sierra Leone is one of the countries in West Africa considered hyperendemic for LF,^2^ with a prevalence of antibodies to the Lassa fever virus (LASV) of 8–52% depending on region.^3,4^ Lassa fever is transmitted to humans via exposure to the excreta of *Mastomys natalensis* (multimammate) rat and also via secondary human-to-human transmission.^5,6^ The majority (about 80%) of early LF infections (within 1 week of exposure) are asymptomatic. Hospitalized patients may present with nonspecific features, such as high fever, general weakness and malaise, sore throat, abdominal pain, headache, nausea and vomiting, diarrhea, productive cough, proteinuria, and anemia.^2,3,7–9^ Clinical presentations typical of LF, such as mucosal bleeding, facial edema, convulsions, confusion or disorientation, pleural effusion, hypotension, elevated transaminases, renal impairment, and coagulation abnormalities, either appear late (after 1 week) or may not appear at all. Death also occurs in severe cases.^3,7,8,10^ Most pediatric cases of LF present as a nonspecific febrile illness.^11^

Health infrastructure in Sierra Leone was badly affected by civil war (1991–2002) and later by the West African Ebola outbreak (2013–2015). The country’s children younger than 5 years (122/1,000 live births), infant (72/1,000 live births), and neonatal (32/1,000 live births) mortality rates remain high, despite significant strides by the government of Sierra Leone (GoSL), over the last decade, to provide free health care to children younger than 5 years and a national commitment to the Millennium and Sustainable Development Goals.^12,13^

Care for patients with LF in Sierra Leone, including pediatric cases, is centered at the Kenema Government Hospital (KGH) in the Eastern Province of Sierra Leone.^14^ Eastern Province, in particular Kenema district, is a Lassa-endemic region, although cases have been reported from 10 of Sierra Leone’s 16 districts.^10,15–17^ There are few studies to date on pediatric LF, making it difficult to fully understand best practices for pediatric LF management.^2,9,11,14,16,18,19^ In West Africa, this dilemma is further challenged because the clinical presentation of LF can be indistinguishable from other viral hemorrhagic fevers (VHFs) and febrile illnesses, such as malaria and typhoid fever. Furthermore, LF can present as a coinfection with other febrile illnesses.^19,20^ This diagnostic challenge impacts at the community and health facility levels, resulting in delays in seeking or receiving treatment and a further risk of community spread and nosocomial infections.^7^ Previous studies have shown a disproportionately negative impact of LF among children and pregnant women.^2,3,8,10^ Peak incidence of antigenemia occurs in early childhood and in adolescence, with high mortality rates in infants younger than 1 year and in early childhood.^10,18,21^ A very severe form of LF, known as “swollen baby syndrome,” characterized by widespread edema, abdominal distention, and bleeding, is mostly seen in neonates and occasionally presents in infants and toddlers.^2,21^

The few studies to date describing clinical characteristics and/or clinical management of pediatric LF cases were predominantly conducted before the civil war and before the development of many of the Lassa diagnostics currently being used at KGH.^15,22^ The 2013–2016 West African and recent Democratic Republic of Congo Ebola outbreaks, however, have brought significant new advances in the treatment and management of Ebola virus disease (EVD), as well as renewed interest in understanding the treatment and clinical management of other VHFs, such as LF. Surveillance and clinical data on childhood LF cases are important for understanding its epidemiology and outcomes as well as informing evidence-based management strategies. The aims of this study were to describe the clinical characteristics of pediatric patients, either suspected or confirmed to have LF, admitted to the KGH LF ward from 2012 to 2018 and to assess factors associated with mortality.

## MATERIALS AND METHODS

### Study design and population.

A retrospective cohort study was conducted using routinely collected programmatic data from the LF ward at KGH. All children (aged 0–18 years) with either antigen-positive LF or who were suspected of having LF on admission to the LF ward between January 1, 2012 and December 31, 2018 were included in this analysis.

### Setting.

**Figure 1.**
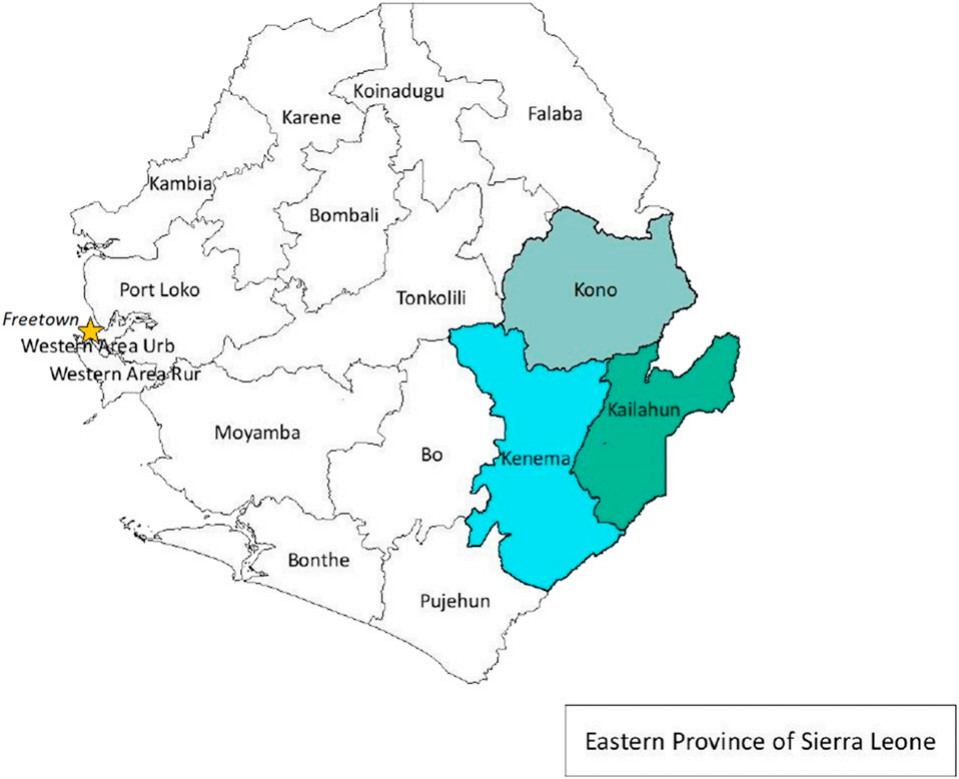
Map of Sierra Leone with Kenema, Kono, and Kailahun districts of Eastern Province highlighted. This figure appears in color at www.ajtmh.org.

This study was conducted at KGH in Kenema district of Eastern Province, which houses the LF Program (Figure 1). Kenema Government Hospital hosts the only dedicated LF treatment unit in Sierra Leone and one of the few LF wards in the world.^3,10,16,17,23^ This treatment unit receives LF-related referrals from facilities across the country. The hospital is equipped with a biosafety level-3 (BSL-3) laboratory for screening suspected cases, as well as a basic clinical laboratory for running tests for patients admitted to the LF ward.^16,23^ Kenema Government Hospital also collaborates with the VHF Consortium, which began in 2010 with support from Tulane University. The goal of the consortium is to conduct state-of-the-art research to better understand important aspects of the immune response and spread of VHFs, including LF and EVD.

### Sample and patient flow of LF suspect cases in children.

When a pediatric patient is suspected of having LF at a peripheral health facility, there are two ways for further diagnostic workup. In one, blood samples are sent to KGH for screening, and if positive, the patient is subsequently referred to KGH and directly admitted to the LF ward. In the other, suspected patients are directly referred to KGH, and diagnostic testing is completed on arrival. Once at KGH, all patients suspected of having LF are maintained within a holding center at KGH until a decision is made about admission to the LF ward.

If a pediatric patient already admitted to the KGH general pediatric ward is later suspected of having LF, the patient is transferred to the holding center, and blood samples are sent to the laboratory for screening. Laboratory samples are typically drawn within 24 hours of the patient’s arrival at KGH or of their being suspected of having LF. All blood samples for suspected LF are screened with a rapid diagnostic test (RDT) for Lassa antigen; ELISA for Lassa antigen, IgM, and IgG; and PCR.

Any child with an RDT positive for LF was admitted to the LF ward. In addition, any child with a high clinical suspicion for LF and an initial positive Lassa IgM test result was admitted to the LF ward, and serial antigen testing was performed. Maximum infection, prevention, and control measures were maintained at all times, including complete use of personal protective equipment. Blood and urine samples were collected on admission for routine laboratory tests, such as complete blood count, urinalysis, liver function tests (LFTs), serum creatinine (Cr) and electrolytes for renal function evaluation, and a coagulation profile.^16,23^ Rapid diagnostic test for malaria and blood smears for malaria parasites were collected as requested by the physician. Intravenous ribavirin was initiated as a 30 mg/kg loading dose within 24 hours of admission and then maintenance dose as follows: 15 mg/kg every 6 hours for 4 days followed by 7.5 mg/kg every 8 hours for 5 days to complete 10 total days of therapy. Further blood samples were collected as per the ward protocol on days 1, 2, 3, 4, 7, and 10. These serial blood measurements helped determine the antigen and antibody response to ribavirin and to monitor serum chemistry levels.

Routine clinical care was provided by a team of healthcare workers dedicated to the LF ward. In addition to the use of intravenous ribavirin, supportive care was provided following the WHO Integrated Management of Childhood Illnesses guidelines (before 2017) or the WHO Emergency Triage Assessment and Treatment guidelines (after 2017).^24,25^ Supportive care typically consisted of administration of intravenous fluids as necessary; broad spectrum antibiotics with either intravenous ceftriaxone or cefotaxime, depending on age; intravenous antimalarial medications if a rapid malaria test was positive; and blood transfusions for patients with anemia. Supportive treatments (such as use of oxygen, nasogastric feeding, and catheterization) and treatment of comorbidities were provided when necessary and if available. Kenema Government Hospital currently has no intensive care units, ventilators, or dialysis capacity and has limited laboratory capacity and no culture capacity for blood, urine, or cerebrospinal fluid. Radiology consists of only X-ray capacity.

### Definitions.

Pediatric LF is defined by the Ministry of Health of Sierra Leone as LF occurring in children aged 18 years of age or younger. A child is considered a suspect case of LF if they have signs and symptoms suggestive of LF (persistent fever of > 38°C despite treatment for malaria or with antibiotics, bleeding from orifices, confusion, or prostration), a history of contact with an LF case, known travel to an LF-endemic area, or an inconclusive RDT for malaria or other illnesses. For the purpose of this analysis, only children who were antigen positive for LF were considered a case of LF. Our outcome variable of interest was determined at hospital day 10 as alive, deceased, or discharged from the LF ward. Patients were discharged from the LF ward when ELISA or PCR results were negative after 10 days. Patients could either be discharged home or transferred to the pediatric ward for further management if there was any comorbidity. A 2-week follow-up visit was scheduled on discharge home, with subsequent follow-up visits scheduled as required.

### Data collection.

Data collection was carried out by trained hospital staff using both paper-based and digital logbooks, various clinical case report forms, and questionnaires, and laboratory data were maintained in both paper-bound laboratory notebooks and electronic format. Demographic information (age, gender, residence, and date of LF diagnosis), clinical presentation (vital signs and other signs and symptoms), laboratory results, administration of ribavirin, and final treatment outcome were collected.

### Data analysis.

Descriptive statistics were used to summarize sociodemographic characteristics using frequencies and proportions (for dichotomous and categorical variables). We compared characteristics of the study cohort by LF antigen status using the chi-squared test or Fischer’s exact test for categorical variables and the Wilcoxon rank-sum test for ordinal/continuous variables. We conducted univariate logistic regression analysis of demographic, clinical, and laboratory variables available at presentation and calculated odds ratios (ORs) and their corresponding 95% CIs to evaluate their association with the hospital outcome of mortality. Multivariate logistic regression was not performed because of our small sample size. Children with missing data for a given variable were excluded from the analysis of that variable. Statistical analyses were conducted using two-sided tests and a 5% significance level. Statistical analysis was performed using Stata version 14.2 (StataCorp LP, College Station, TX).

### Ethical considerations.

The Sierra Leone National Ethics and Scientific Review Committee (August 13, 2019 to August 12, 2020), the Tulane University Institutional Review Board (Ref# 140674), and the Vanderbilt University Institutional Review Board (IRB# 201010) approved this analysis.

## RESULTS

Between January 1, 2012 and December 31, 2018, a total of 292 children aged 0–18 years were evaluated for possible LF. Fifty-seven (20%) children were classified as patients with LF based on antigen testing. Among the children who were antigen-positive for LF, there was a significantly higher proportion (63%, *P* = 0.031) of males than children who were antigen-negative. Overall, 60% of children in our cohort were aged 0–9 years. Seventy-nine percent (79%) of children suspected of LF were admitted to KGH in a 3-year period from 2012 to 2014, followed by a dramatic reduction in the absolute numbers of admissions in the subsequent 4 years of analysis. Regardless of LF antigen status, mortality was high among our cohort of children (21%). The case fatality rate (CFR) was significantly higher (63% versus 11%; *P* < 0.001) in the children who were LF antigen-positive than children who were LF antigen-negative (Table 1).

**Table 1:** Characteristics of confirmed and suspect pediatric LF patients evaluated by the Kenema Government Hospital, LF Program (2012–2018)

	Lassa confirmed antigen-positive (*n* = 57)	Lassa suspect antigen-negative (*n* = 235)	Total (*n* = 292)	*P*-value*
Gender				
Male	36 (63%)	111 (47%)	147 (51%)	0.031
Female	21 (37%)	124 (53%)	145 (49%)	
Age (years)				
0–4	23 (40%)	75 (32%)	98 (34%)	0.138
5–9	17 (30%)	60 (26%)	77 (26%)	
10–14	7 (12%)	44 (18%)	51 (17%)	
15–18	10 (18%)	56 (24%)	66 (23%)	
Year				
2012	16 (28%)	83 (35%)	99 (34%)	0.466
2013	20 (35%)	79 (34%)	99 (34%)	
2014	9 (16%)	24 (10%)	33 (11%)	
2015	3 (5%)	4 (2%)	7 (2%)	
2016	5 (8%)	12 (5%)	17 (6%)	
2017	2 (4%)	16 (7%)	18 (6%)	
2018	2 (4%)	17 (7%)	19 (7%)	
Outcome				
Death	36 (63%)	25 (11%)	61 (21%)	< 0.001
Discharge	20 (35%)	102 (43%)	122 (42%)	
Transferred	0 (0%)	16 (7%)	16 (5%)	
Not admitted to LF ward	1 (2%)	90 (38%)	90 (31%)	
Unknown	0 (0%)	2 (1%)	3 (1%)	

LF = Lassa fever.

* Chi-square and Fisher’s exact used for categorical variables. Wilcoxon rank-sum for ordinal/continuous variables.

### Clinical features.

Overall, 37% of children found to be LF antigen-positive had a temperature of 38°C or higher at admission (data not shown). Among LF antigen-positive children, cough (86%), vomiting (75%), and headache (74%) were the most common presenting symptoms, followed by sore throat (58%), pain (58%), head/neck edema (56%), and diarrhea (56%). Unexplained bleeding was reported by 48% of patients and confusion by 35% (Table 2).

**Table 2:** Clinical symptoms of pediatric antigen-positive Lassa fever patients (2012–2018)

*N* = 57	*N*	Survived (*n* = 21) median (IQR)	*N*	Died (*n* = 36) median (IQR)	*P*-value*
Vitals					
Temperature (°C)	21	37.7 (37, 38.4)	32	38.0 (36.8, 38.7)	0.934
Oxygen saturation (%)	15	98 (92, 99)	21	95 (91,98)	0.042
Symptoms†		(%)		(%)	
Unexplained bleeding	5	24	19	53	0.033
Pain	13	62	20	56	0.640
Conjunctivitis	6	29	13	36	0.560
Head/neck edema	11	52	21	58	0.662
Rash	3	14	1	3	0.136
Headache	15	71	27	75	0.768
Cough	17	81	32	89	0.449
Sore throat	12	57	21	58	0.930
Vomiting	14	67	29	81	0.240
Diarrhea	10	48	21	58	0.433
Convulsions	7	33	14	39	0.675
Confusion (altered sensorium)	3	14	17	47	0.012
Jaundice	1	5	3	8	1.000
Treatment					
Received ribavirin	15	72	23	64	0.916
Did not receive	3	14	5	14	
Missing	3	14	8	22	

°C = degrees Celsius; IQR = interquartile range.

* Wilcoxon rank-sum, Fisher’s exact, and chi-square tests.

† Symptoms are not exclusive. Could add up to more than 100%.

Among the 57 children with LF antigen-positive results, 38 (67%) received ribavirin, of which 23 (64%) died. Receipt of ribavirin was not significantly associated with survival among LF antigen-positive children (*P* = 0.916) (Table 2).

### Risk factors for death.

In univariate analysis, children presenting with a symptom of unexplained bleeding had a more than 3-fold higher odds of death (OR: 3.58; 95% CI: 1.08–11.86; *P* = 0.040). In addition, children who presented with confusion (altered sensorium) had a more than 5-fold higher odds of death (OR: 5.37, 95% CI: 1.34–21.48; *P* = 0.020) (Table 3). Lower oxygen saturation was also shown to be significantly associated with mortality (*P* = 0.042) (Table 2).

**Table 3:** Univariate analysis: Factors associated with mortality among antigen-positive pediatric patients in Kenema, Sierra Leone

*N* = 57	Odds ratio (95% CI)	*P*-value
Gender		
Female	Ref	0.130
Male	0.39 (0.12–1.30)	
Age (per 1-year increase)	1.05 (0.95–1.16)	0.340
Symptoms*		
Unexplained bleeding	3.58 (1.08–11.86)	0.040
Confusion (altered sensorium)	5.37 (1.34–21.48)	0.020
Head/neck edema	1.27 (0.43–3.76)	0.660
Laboratory values		
BUN lower quartile (1.8–3.6 mmol/L)†	Ref	
BUN upper quartile (14.6–39.2 mmol/L)	20.1 (0.93, 432.75)	0.055
Cr lower quartile (22–44 mmol/L)†	Ref	
Cr upper quartile (144–1,057 mmol/L)	31.6 (1.37, 725.23)	0.031
ALT lower quartile (14–220 U/L)†	Ref	
ALT upper quartile (1,326–1998 U/L)	361 (6.47, 20,143.55)	0.004
Bilirubin lower quartile (7–10 mmol/L)	Ref	
Bilirubin upper quartile (33–77 mmol/L)	4.80 (0.68, 33.80)	0.115
Sodium lower quartile (113–124 mmol/L)	Ref	
Sodium upper quartile (138–166 mmol/L)	14.0 (1.54, 127.23)	0.019
Potassium lower quartile (3–4.3 mmol/L)	Ref	
Potassium upper quartile (6.5–8.6 mmol/L)	21.0 (1.50, 293.25)	0.024
Treatment*		
Received ribavirin	0.71 (0.22–2.27)	0.560

ALT = alanine aminotransferase; BUN = blood urea nitrogen; Cr = Creatinine; mmol/L = millimole/liter.

* Comparison of each symptom or treatment versus those without the symptom or treatment as reference value.

† Firth logistic regression.

Among the 57 children with LF antigen-positive results, those who died had higher serum blood urea nitrogen (BUN) (8.6 versus 4.3 millimole/liter [mmol/L]; *P* = 0.014), alanine aminotransferase (ALT) (1255 units/liter [U/L] versus 220 U/L; *P* = 0.001), and potassium (5.95 versus 4.35 mmol/L; *P* = 0.003) (Table 4). Children in our cohort whose serum Cr levels fell within the highest quartile of values (144–1,057 mmol/L) had a more than 30-fold (OR: 31.6; 95% CI: 1.37–725.23; *P* = 0.031) odds of death than children whose serum Cr levels were within the lowest quartile of values (22–44 mmol/L). In addition, children with serum ALT values within the highest quartile of values (1,326–1998 U/L) had more than 300-fold (OR: 361; 95% CI: 6.47–20,143.55; *P* = 0.004) higher odds of death than children in the lowest quartile of values (14–220 U/L) (Table 3). Finally, children with serum levels of sodium and potassium in the highest quartile of values at presentation had a 14-fold (OR: 14.0; 95% CI: 1.54–127.23; *P* = 0.019) and 21-fold (OR: 21.0; 95% CI: 1.50–293.25; *P* = 0.024) higher odds of death, respectively, than children in the lowest quartile.

**Table 4:** Laboratory results for children who were Lassa fever antigen–positive

*N* = 57	*N*	Survived (*n* = 21) median (IQR)	*N*	Died (*n* = 36) median (IQR)	*P*-value*
Laboratory test with normal values					
Blood urea nitrogen (2.9–7.9 mmol/L)	15	4.3 (3.2,6.3)	20	8.6 (3.8, 20.55)	0.014
Creatinine (53–106 mmol/L)	14	45.5 (27.0, 69.0)	20	95.5 (63.0, 296.5)	0.004
Alanine aminotransferase (10–48 units/L)	17	220.0 (56.0, 817.0)	19	1,255.0 (570.0,1618.0)	0.001
Total bilirubin (3–27 mmol/L)	16	10.5 (9.5,17.5)	23	16.0 (11.0,33.0)	0.149
Sodium (128–145 mmol/L)	16	126.5 (122.0,132.0)	22	133.5 (126.0,138.0)	0.017
Potassium (3.6–5.1 mmol/L)	14	4.35 (4.1,4.6)	16	5.95 (4.9, 7.15)	0.003

mmol/L = millimole/liter.

* Wilcoxon rank-sum test.

## DISCUSSION

In our study, we set out to provide a description of current clinical characteristics seen in patients suspected and diagnosed with LF admitted to KGH in Eastern Province, Sierra Leone, and to explore potential factors associated with mortality in children with LF antigen–positive results. We report a disease positivity rate of 19.5% (57/292) among children suspected of LF and a CFR of 63% (36/57) among our LF antigen–positive cohort. The CFR reported in our pediatric cohort is much higher than the CFR of 29.2% reported in a recent pediatric cohort from Ebonyi, Nigeria,^26^ and higher than the overall CFR of 15% generally reported by the WHO for hospitalized patients with LF (both adult and children).^27^ Although the prevalence and CFR for pediatric LF are still not well described, it has been reported that asymptomatic or mild disease represents approximately 80% of all LF infections.^28^ As such, the differences in the CFR in our cohort may represent a sicker patient population presenting for admission to the KGH LF ward compared with what has been reported elsewhere. Furthermore, four major genetically distinct viral lineages have been described, of which three are endemic to Nigeria and the fourth is endemic to the rest of West Africa. Variable pathogenicity across LF lineages may contribute to differences in the CFR and needs to be explored further.^28,29^

The nonspecific signs and symptoms of LF have been widely studied in adults. Clinical presentations in children with LF and other VHFs, though less documented, are also described as nonspecific and highly diverse. Consistent with a 1986 study conducted in Sierra Leone,^14^ we found that 60% of our LF antigen–positive cohort were between 0 and 9 years of age. However, in contrast to previous reports, we found a higher proportion of our cohort to be male.^14,26^ As with previous descriptions of pediatric LF, cough, vomiting, headache, low oxygen saturation, and temperature ≥ 38°C were common initial clinical presentations in our cohort.^2,14,19^ Most of these symptoms have been described by the WHO as minor criteria for LF and in some studies as typical of “early presentation” with a good clinical prognosis when treatment with ribavirin is started early. Interestingly, our study also documented children with clinical presentations at admission that are generally considered as major criteria for LF or that previous studies have classified as “late presentations.”^2,10,11,19,30^ Confusion (altered sensorium), unexplained bleeding, and head/neck edema were common among our cohort of children with LF antigen–positive results who died. Univariate analyses showed a statistically significant higher OR of mortality in children with confusion and unexplained bleeding. It is important to point out that we are unable to determine the length of symptoms of these children before their presentation to KGH. More severe presenting symptoms may represent a culture of delayed health-seeking behavior among our cohort, resulting in patients being generally sicker at the time of presentation to the hospital. Nonetheless, we feel that these symptoms are important prognostic factors for antigen positivity and mortality in this age-group. Although protocols at KGH recommend simultaneous testing for potential comorbid conditions such as malaria, typhoid, HIV, and tuberculosis, documentation of other laboratory testing performed in our cohort was limited and incomplete. The retrospective nature of this analysis does not elucidate whether this incompleteness was due to unperformed tests or if the results of these tests were inappropriately documented.

Much of our understanding of the pathogenicity of LASV comes from studying animal models of the disease.^28,31^ Based on these models, we know that LASV infection targets macrophages and dendritic cells, resulting in myeloid cell dysregulation, subsequent spread to multiple organ systems, followed by host inflammatory and vasodilatory processes. Systemic spread has been shown to involve the liver, spleen, kidneys, pancreas, uterus, gonads, and epithelial tissue, among others.^28^ Human studies have shown that abnormalities of both liver and renal function have been associated with LASV infection and other VHFs (though not widely studied in pediatric populations).^2,15^ We found that elevated serum levels of ALT, a biomarker for liver function and damage, and elevated serum levels of BUN and Cr, both biomarkers of renal function and damage, were associated with significantly higher odds of death in our pediatric cohort. Primate studies have shown that high viral titers can be found in the liver and have contributed to our recognition that it is a site of major viral replication, exemplified by a significant rise in both ALT and aspartate aminotransferase (AST).^32^ In our cohort of patients with LF antigen–positive results, elevated serum ALT at hospital presentation was extremely common. Similarly, we found that elevated serum BUN and Cr at presentation were significantly associated with mortality. Although not all our patients with LF antigen–positive results who died had elevated serum BUN and Cr, those with values in the highest quartile had a 20-fold and 30-fold higher odds of death, respectively. This finding is consistent with what is known from postmortem examinations of LF patients in which tubular necrosis, interstitial nephritis, and glomerular sclerosis were common findings.^33,34^ Both elevated LFTs (ALT and AST) and elevated BUN and Cr should be red flags to clinicians as potentially prognostic of poor outcomes.

It is not clear why only 66% (38/57) of our cohort with LF antigen received ribavirin or why ribavirin was not associated with better survival. In the recent pediatric report from Ebonyi, Nigeria, only 19% of LF-positive children received ribavirin died.^26^ Unfortunately, incomplete collection of data on length of symptoms before admission, duration of ribavirin administration, and justification for deferral of ribavirin does not allow us to comment on this.

Electrolyte abnormalities in children with LF have not previously been described in detail. The lower serum sodium levels of children who survived could be attributed to fluid loss from vomiting; however, a higher number of children in our cohort who died presented with vomiting, although their serum sodium levels were within normal limits. Serum potassium levels were elevated in children who died. Possible contributing factors include aggressive correction of dehydration with potassium-containing fluids (Ringer’s lactate) following diarrhea and vomiting and kidney disease.

Our study analyzed data from a total of 292 children admitted to the LF ward at KGH over a period of 7 years (January 1, 2012 and December 31, 2018). These data are important because they give insights into the yearly variability of children evaluated for admission to the KGH LF ward. Although we do not fully understand the drop-off in cases seen over time, there are several possible explanations. First, significant work was carried out beginning in 2010 to establish and implement a data capture and management system at the KGH LF ward, which allowed for more efficient and rapid patient triaging and diagnostics, thus improving decision-making related to admissions into the LF ward.^23^ In addition, higher admission numbers early in our period of analysis could be due to an increase in the number of children presenting to healthcare facilities in Sierra Leone following the launch of a Ministry of Health free healthcare initiative in 2010.^10^ One explanation for the decrease in admission numbers seen following 2013 may be the onset of the 2013–2016 West African Ebola outbreak. During this crisis, databases and surveillance structures that were originally designed for capturing LF cases were reappropriated for identification of EVD cases. In addition, this period saw heightened levels of mistrust in the healthcare system and a fear that hospitals were a place where people went to die.^35–37^ Another possible cause for the decrease in admissions later in the study period could be improved diagnostic testing (RDT and ELISA), as later in the study period, children suspected of LF were only admitted to the ward after LF testing, whereas the previous criterion for admission was suspicion of LF.^10,23^

This study has several limitations. First, despite improvements in data management at KGH over the past decade, data collection remains incomplete, especially during the 2013–2016 West Africa Ebola outbreak. Certain patient characteristics such as weight, immunization history, nutritional and past medical history, other comorbid conditions, and vital signs that are key in the management of pediatric cases were not always captured. We found that certain vital signs were not consistently recorded to include in the analysis. Another limitation was our small sample size of LF antigen–positive children, which limited our ability to conduct multivariable analyses that adjusted for potential confounders. Our study was limited to patients admitted to KGH, with limited data documenting from where these patients were referred. As such, we are limited in our ability to generalize outside of Kenema. Finally, there were several limitations to our interpretation of laboratory data. There is limited information in the literature on the standard reference laboratory values for renal and LFTs in children. Reference values used at KGH were those for adults, which could lead to interpretation bias. Furthermore, although serial laboratory measurements are typically carried out for patients admitted to the LF ward, only laboratory results at presentation were completely recorded for the purpose of this analysis, limiting our ability to analyze these values over time. We report elevations in ALT seen in patients with LF antigen–positive results who died, but there are no data on AST. The KGH LF program uses the Piccolo Xpress point of care chemistry analyzer, which includes a liver panel. One of the limitations of this machine is that moderate and severe hemolysis in the sample can negatively affect the results for AST.^38^ In our cohort, we found that AST results were not consistently recorded to include in this analysis. Discussions with our laboratory technicians concluded that their lack of confidence in the AST readouts, resulting from hemolysis, is the reason these values were not recorded.

In conclusion, our findings provide insights into the clinical presentation of pediatric LF and potential associations with mortality. A high index of suspicion is needed for the management of these cases. More evidence-based, high-quality research in creating predictive algorithms of antigen-positivity and hospital outcomes is needed in the management of LF and, broadly, other VHFs.
